# A Novel Polar Copolymer Design as a Multi-Functional Binder for Strong Affinity of Polysulfides in Lithium-Sulfur Batteries

**DOI:** 10.1186/s11671-017-1948-5

**Published:** 2017-03-16

**Authors:** Yu Jiao, Wei Chen, Tianyu Lei, Liping Dai, Bo Chen, Chunyang Wu, Jie Xiong

**Affiliations:** 1 School of Applied and Chemical Engineering, Xichang College, Xichang, 615053 China; 2 State Key Laboratory of Electronic Thin Films and Integrated Devices, University of Electronic Science and Technology of China, Chengdu, 610054 China; 3 Institute of Microelectronics of Chinese Academy of Sciences, Beijing, 100029 China

## Abstract

High energy density, low cost and environmental friendliness are the advantages of lithium-sulfur (Li-S) battery which is regarded as a promising device for electrochemical energy storage systems. As one of the important ingredients in Li-S battery, the binder greatly affects the battery performance. However, the conventional binder has some drawbacks such as poor capability of absorbing hydrophilic lithium polysulfides, resulting in severe capacity decay. In this work, we reported a multi-functional polar binder (AHP) by polymerization of hexamethylene diisocyanate (HDI) with ethylenediamine (EDA) bearing a large amount of amino groups, which were successfully used in electrode preparation with commercial sulfur powder cathodes. The abundant amide groups of the binder endow the cathode with multidimensional chemical bonding interaction with sulfur species within the cathode to inhibit the shuttling effect of polysulfides, while the suitable ductility to buffer volume change. Utilizing these advantageous features, composite C/S cathodes based the binder displayed excellent capacity retention at 0.5 C, 1 C, 1.5 C, and 3 C over 200 cycles. Accompany with commercial binder, AHP may act as an alternative feedstock to open a promising approach for sulfur cathodes in rechargeable lithium battery to achieve commercial application.

## Background

The lithium-sulfur (Li-S) rechargeable battery cells offering a theoretical cathode specific capacity of 1675 mAh g^−1^, which is five times higher than those commercial lithium ion batteries (LiCoO_2_ and LiFePO_4_), have been applied in a variety of the most promising energy storage devices to address the increasing energy storage demands for various technological applications [1–3]. Unfortunately, despite its considerable advantages, its practical use has been frustrated by several problems [4–6]. (1) The sulfur is low electron conductivity (5 × 10^−30^ S cm^−1^ at 25 °C), which generally causes low utilization of active materials. (2) Large variation in volume occurs during charge-discharge cycling, corrode cathode where they are not recycled on charge. (3) “Shuttle effect”, another major problem caused by the high dissolution of the discharge/charge, intermediates in organic electrolytes. Polysulfides dissolve into the electrolyte and penetrate through the separator to the lithium metal anode and then they are reduced to solid precipitates (Li_2_S), leading to quick capacity decay with the loss of active materials and an additional problem of low Coulombic efficiency in a rechargeable Li-S battery [7–10]. Although various approaches have been employed to overcome this problem [11–14], such as N-doped materials [15–17], carbon-based materials [18], conductive polymers [19, 20], metal oxides [21–23], and transition metal disulfides [24]. None have proven commercially viable due to its high cost and not suitable for large scale manufacturing.

The binder is an important ingredient in Li-S battery, it functions to bond and keep the active materials in the electrode, to ensure well electrical contact between the active materials and conductive carbon, as well as to link the active materials with the current collector [24–28]. In particular, the recent investigations on silicon anodes have revealed that ideal binder should not only able to adhesion strength and ductility with inexhaustible tolerance of large volume change and still physically and/or chemically trap capacity to attain high initial reversible capacity and excellent cycle ability. Polyvinylidene fluoride (PVDF) is widely used as a conventional binder for Li-S batteries [28]. However, due to the linear-molecular structure, PVDF just play a role of physical adhesion, enabling the mechanical linkage of the active materials with additives, the function will fade with time when there is no bonding between those polymers and the carbon substrate, resulting in the vexed problem that polysulfide dissolved in the aprotic electrolyte. Therefore, new functional binder is the urgent needs to make up for the deficiencies of PVDF. Thus it can be seen crosslink structured binder for further improvement in the cycle life is constructed with the increased number of active sites between polysulfides and binder. Recent investigations shown that functional materials endued with amine groups have been viewed as an ideal hunter to absorb polar lithium sulfides and nonpolar carbon surface, which effectively prevents loss of active mass during cycles [29, 30].

Hence, in this paper, we introduced a multi-functional AHP binder with plenty amide groups as an efficient binder for Li-S batteries. Strong interactions between discharge products polysulfides are created throughout the cathode by the unique amide/amino crosslink structures of designed binder to buffer the shuttle effect of sulfur cathodes. Unlike conventional polymeric binders (PVDF), obvious superiority of our design is the binder with interconnected polar structure to form a stable electrode, and exhibit ductile architecture, resulting in a marked improvement of conventional C/S cathode in cycle life. It is noteworthy that the presented strategy is not engineered in any specialized manner, thus making the process commercially viable.

## Methods

### Synthesis of the AHP Binder

Ethylenediamine (EDA), hexamethylene diisocyanate (HDI), and *N*,*N*-dimethylformamide (DMF) were purchased from Aladdin and used as received. The novel AHP binder was prepared by a copolymerize process using EDA (10 mmol) and HDI (5 mmol) in DMF solvent with high-speed magnetic stirring for 4 h at 60 °C. Then the product was uniformly dispersed in DMF solution with a mass ratio of 1 mg per 10 uL solvent.

### Characterization

X-ray photoelectron spectroscopy (XPS, Kratos Axis Ultra Dld, Japan) was used for elemental analysis and chemical bonding information after synthesis of EDA and HDI. Scanning electron microscope (SEM) was used to observe the surface topography of S cathodes with different binder before and after cycle.

### Preparation of S@AHP Cathodes and Electrochemical Measurements

Bulk sulfur (Alfa Aesar, 043766) and acetylene black (Hefei Kejing Materials Technology Co., Ltd) with a mass ratio of 6:4 was ball milled for 60 min at 300 rpm. The obtained mixture was then heated at 210 °C for 12 h to encapsulate sulfur in the acetylene black. After cooling to room temperature, the C/S composite was obtained. Then the thermogravimetric analysis (TGA, SDT 2960, USA) was performed on an SDT 2960, TA Instruments to confirm the mass of sulfur. Typically, the preparation of electrodes and battery assembly were that the electrodes from the C/S composite were prepared by making slurry of C/S and AHP binder in a mass ratio of 8.5:1.5 in DMF solvent, respectively. The slurry was then casted on the surface of Al foil and dried under vacuum at 60 °C overnight. Electrodes contained approximately 0.5 mg of sulfur per square centimeter, and 30 uL electrolyte was used in a coin cell. For comparison, C/S cathodes with various binders were prepared using PVDF and PTFE (both from Hefei Ke Jing Materials Technology Co., Ltd) instead of AHP by similar route.

The electrolyte was 1 M LiTFSI dissolved in a mixture of 1,3-dioxolane (DOL) and 1,2-dimethoxyethane (DME) (1:1 v/v) with 1wt% lithium nitrate (LiNO_3_) as additive. Cells were assembled in an argon-filled glove box, and the electrochemical test of discharge-charge properties and cyclic voltammetric were tested on the CT2001A cell test instrument (Wuhan LAND Electronic Co., Ltd) and CHI660E (Shanghai Chenhua instrument Co., Ltd) electrochemical workstation, respectively.

## Results and Discussion

**Fig. 1:**
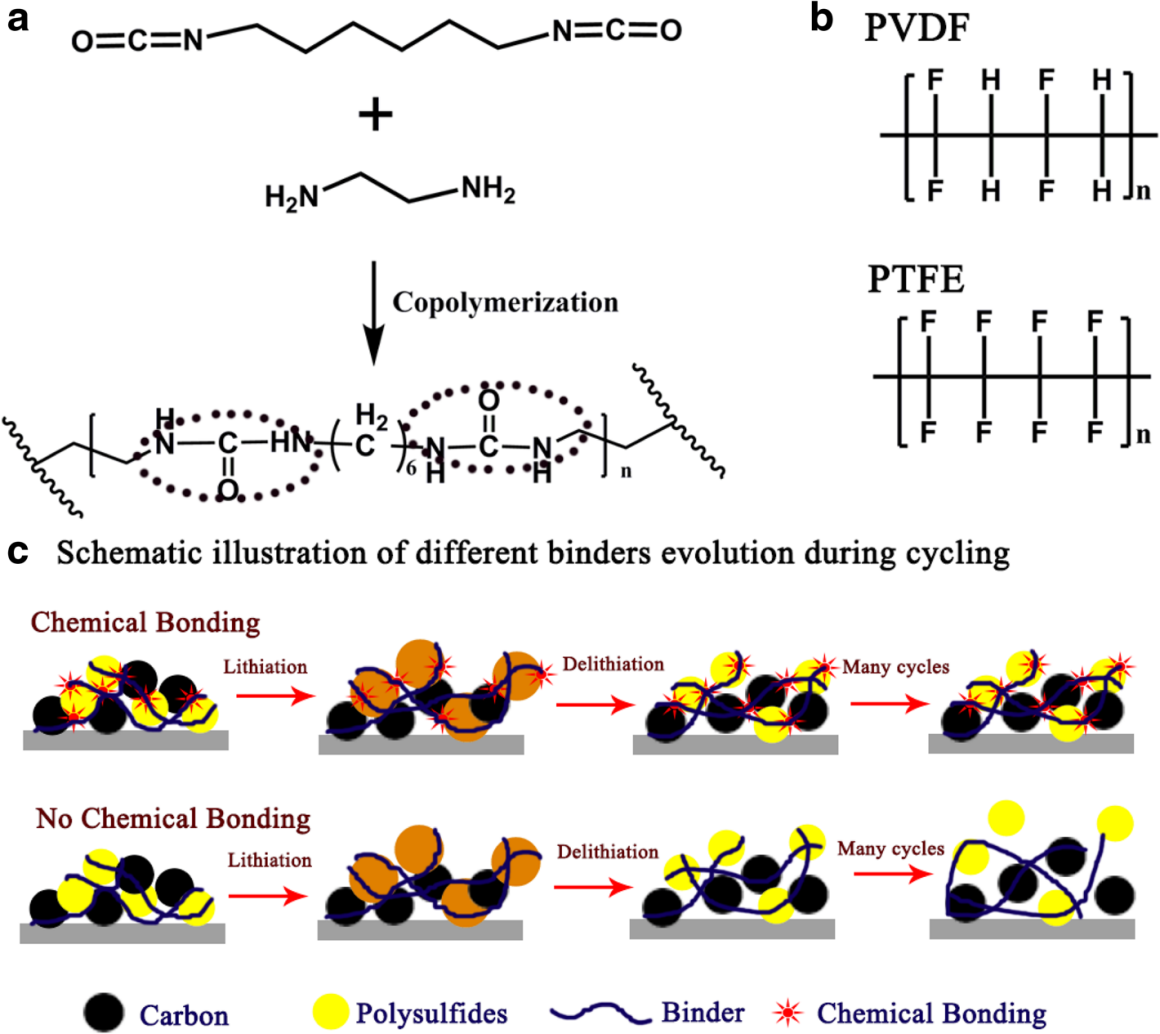
**a** Synthesis scheme of AHP copolymerize EDA with HDI in DMF solution. **b** Commercial binders of PVDF and PTFE. **c** Schematic illustration of different binder evolution during cycling

Figure 1a shows the design concept and synthesis schematic of the polar AHP binder that the linear HDI, acting as a bridge, grafts with EDA by the undiscriminating reactions between amine groups in EDA and isocyanates, resulting in (or forming) a much active site structure of AHP. Upon polymerization, the AHP structure incorporating a series of amide groups enable the binder to attain strong binding energy with polysulfides [31]. Comparing with commercial binders (such as PVDF, PTFE, Fig. 1b), significant advantages were introduced to the novel binder for Li-S battery. Polar group of amide was incorporated to strong anchor Li_2_S_n_ species, which is thought to have a strong affinity to lithium polysulfides, effectively keeping them within the cathode region and thus improving the electrochemical stability of the Li-S battery [32–35].

To better understand the effect of the mechanical and chemical characteristics among electrode components, the binding structural viewpoint of S with AHP and commercial linear binders (such as PVDF, PTFE) are expected to be different (Fig. 1c). During the discharge process (Li insertion), owing to the formation of insulating Li_2_S on the carbon matrix, linear PVDF or PTFE are forced to mechanical stretch or moved by the expanded polysulfides. The volume density of Li_2_S (1.67 g/cm^3^) is much lower than that of S (2.03 g/cm^3^), which would cause a volume expansion of the S cathode of ∼22% compared to their initial state during the whole discharge process. Upon charging (Li desertion) in the same cycle, the polysulfides shrink back to their original state; the linear binders, however, cannot fully follow the shrinkage of polysulfides, thus leading to an inevitably contact loss of electroactive materials from the carbon matrix, coupled with polysulfides dissolution, result in inferior performance of most sulfur-carbon composites. This issue becomes more prominent over extended cycling. On the contrary, AHP have plentiful amide side groups physically/chemically entangled to grasp polysulfides, leading to reinforced binding ability with polysulfides via hydrogen bonding [29, 30]. Thus, in this polymeric AHP binder provides multidimensional noncovalent interactions with the polysulfides surfaces through the amide groups. These interactions not only allow the AHP binder to accommodate the massive volume expansion of S cathodes during discharge process but also keep the polysulfides-binder interactions even during charge process.

**Fig. 2:**
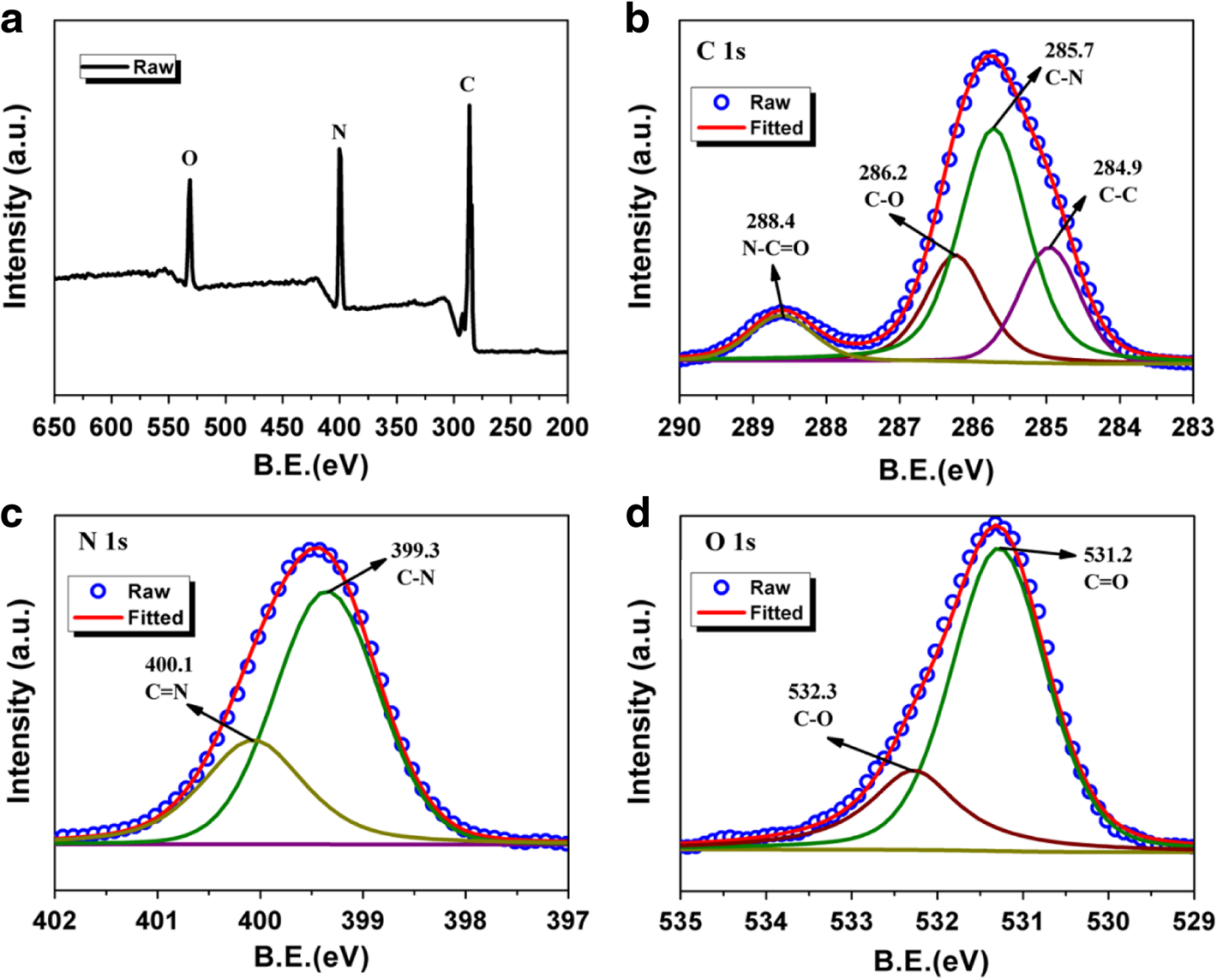
XPS scanning spectra for **a** AHP binder and **b**, **c**, **d** high-resolution C 1s , N 1s, and O 1s spectrum of AHP, respectively

The AHP binder was covalently cross-linked after the undiscriminating reactions of EDA with HDI. Therefore, to observe the possible mechanism of the discovered reaction between bio-inspired EDA and HDI, X-ray photoelectron spectroscopy (XPS) survey scanning spectra were utilized to prove the possible chemical characterization of the covalently binder, as presented in Fig. 2. Definitely, deconvolution of the N 1s signal reveals peaks for both amine (399.3 eV) and amide (400.1 eV) groups can be found, undoubtedly emerges from AHP binder, indicating that EDA has taken part in the reaction with HDI. It is to imply that after reaction, the amino groups were also retained and form polar amide groups. Besides, the C 1s signal can be well resolved corresponding to C–N amide bond at 288.4 eV and C–NH_2_ bond at 285.7 eV. Furthermore, the C=O (531.2 eV) 1s peak was observed (or collected) in the wide O 1s spectra. All of the results have provided hard evidence that the polymerization with EDA and HDI had occurred to formamide groups, which are benefitted to capture polysulfides.

**Fig. 3:**
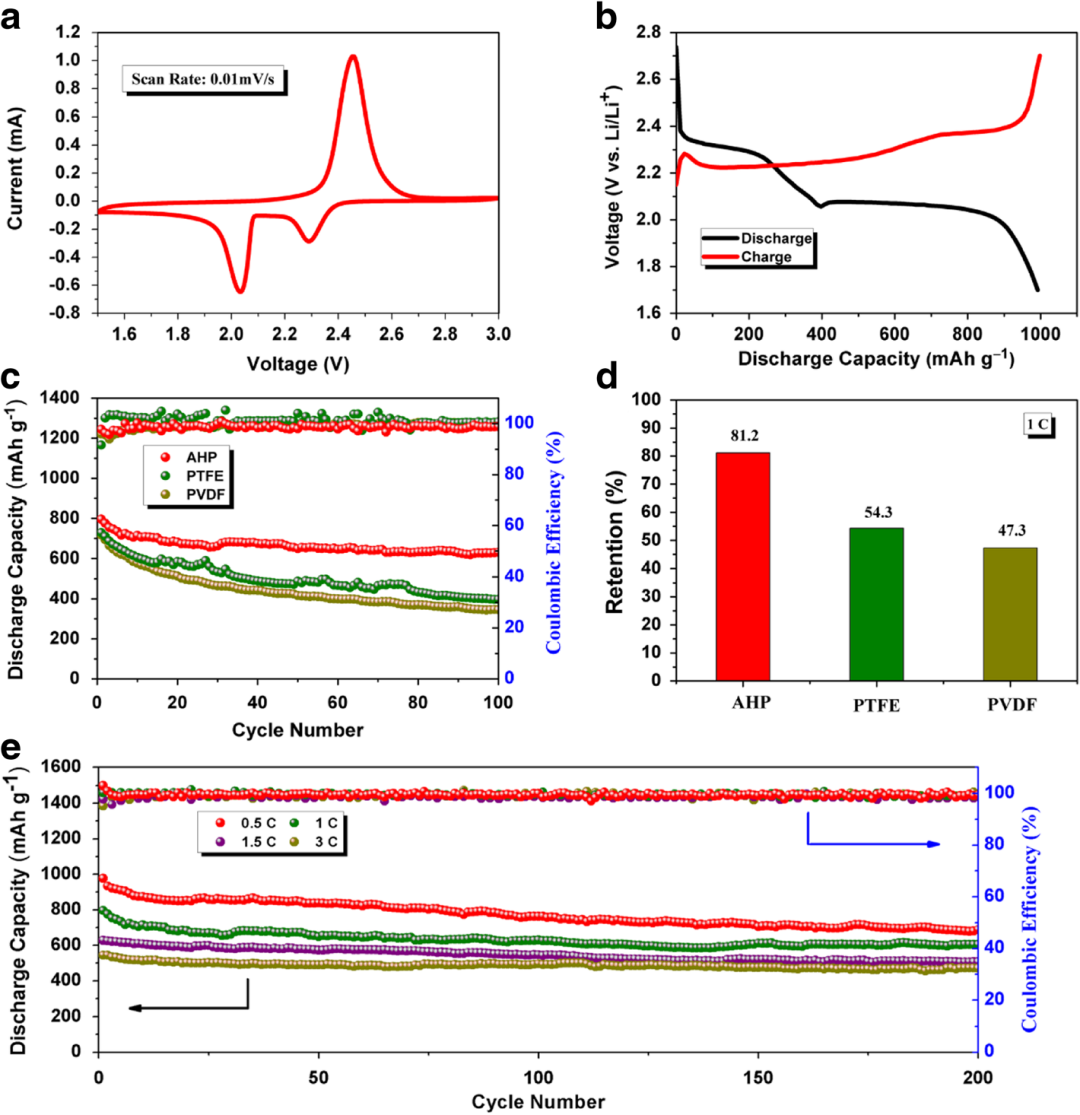
Electrochemical measurement of AHP binder. **a** The CV curve of S@AHP electrodes at a scan rate of 0.01 mV S^−1^ between 1.5–3.0 V. **b** (Dis) charge voltage profiles at 0.5 C (1C = 1672 mA/g). **c**, **d** The comparison performance between S@AHP, S@PTFE, and S@PVDF at a rate of 1 C, and the retention over 100 cycles. **e** Long-term cycling performance of S@AHP cathodes between 1.7–2.8 V with cycling performance and Coulombic efficiency at different current rates (0.5 C, 1 C, 1.5 C, and 3 C)

In order to explore the electrochemical performance of S electrodes with the AHP binder, a series of electrochemical tests were carried out. CR2025 type coin cells were fabricated using lithium foil as the counter electrode. Figure 3a shows cyclic voltammetry (CV) of the C/S/AHP composite cathode at a scan rate of 0.01 mV S^−1^ between 1.5 and 3 V (vs Li/Li^+^). According to the multiple reaction mechanism between S and Li, two cathodic peaks are clearly observed: one is located at ~2.30 V attributing to the transformation of S_8_ to long-chain Li_2_S_n_ (4 ≤ *n* ≤ 8), and the other at ~2.05 V was ascribed to the further reduction of S to form low-order Li_2_S_n_ (*n* < 4), and finally Li_2_S. Anodic peaks are caused by the decomposition of Li_2_S and corresponding to the reverse process of the transformation of sulfur species to Li_2_S_n_. Consistent with the CV analysis above, Fig. 3b shows the typical two-plateau charge/discharge profile of the C/S/AHP composite cathode at a current rate of 0.5 C, which could be assigned to the formation of long-chain polysulfides (high plateau) and short-chain polysulfides (low flat plateau), which was typical charge/discharge profile of Li-S cells.

Long-term cycling and well stability is the first goal of a commercial battery, the electrochemical stability of the C/S/AHP composite cathode was investigated by testing under 1 C for 100 cycles, compared with similar electrodes using PTFE and PVDF as binders. The cycle life, discharge capacity, and Coulombic efficiency of electrode with AHP as binder were significantly better than those with PTFE and PVDF. As shown in Fig. 3c, after 100 cycles, the capacity of AHP binder stabilizes at 628 mAh g^−1^ with 81.2% retention (Fig. 3d) at 1 C. It is shown, in our work, the capacities of the conventional PTFE and PVDF binders drop very fast. The discharge capacity of S@PTFE started at capacity of 728 mAh g^−1^ but degraded severely to 395 mAh g^−1^ after the same number of cycles, which corresponds to 54.3% capacity retention, and the PVDF binder dropped more severely with 47.3% capacity retention. The S@AHP electrode exhibits the best cycling performance compared with common binders in Li-S batteries when the commercial sulfur powder is taken as active material. The stable cycling performance and high Coulombic efficiency (99%) imply that the AHP binder is benefit to confine polysulfides in the eletrode. Over 200 cycles, the reversible capacities of the C/S@AHP electrode at different rates of 0.5 C, 1 C, 1.5 C and 3 C also show excellent stability (Fig. 3e). Apparently, the enhanced reversibilityof AHP binders contribute a lot to cyclic performance of S electrode a possible mechanism is that the plentiful amide groups could efficiently inhibit the leakage of polysulfide during cycling. The much improved performance of the C/S@AHP electrode was due to the fact that the polar amino group of the binder provides the strong affinity to absorb lithium polysulfide intermediates, resulting in enhanced cycling performance [29, 30].

**Fig. 4:**
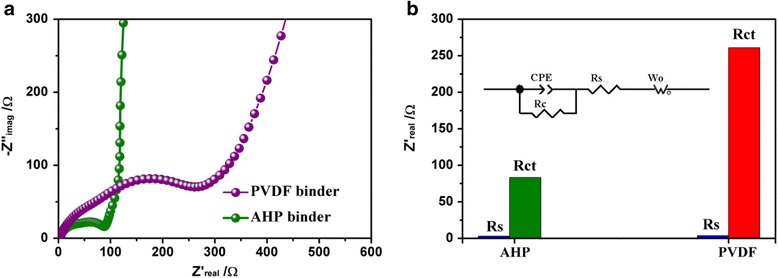
**a** AC impedance spectroscopy data of a lithium cell with the AHP and PVDF composite cathodes, (**b**) the resistance of AHP and PVDF binders according to the AC impedance spectroscopy

The electrochemical impedance spectroscopy (EIS) measurements of AHP and PVDF binder were conducted within the frequency range between 0.1 Hz and 1 MHz. The Nyquist plot, presented in Fig. 4a, is composed of a depressed semicircle at high frequencies that corresponds to the solution resistance (R_s_) and the interfacial charge transfer resistance (R_ct_), which is related to the electrochemical activities of the composites [34–36]. The short, inclined line in the low-frequency region is associated with a semi-infinite Warburg diffusion process (W) of soluble lithium polysulfide in the electrolyte. According to the quantitative analysis (Fig. 4b), the changes between S@AHP cathode and S@PVDF cathode in Rs are not significant. In contrast, the variation of R_ct_ is strongly associated with charge transfer of cathodes [36–38]. This results are benefited to the excellent adhesion of AHP binder that strong and multidentate interactions with active materials were created that can effective promotion contact between S and the carbon matrix and is conducive to the charge transfer, resulting in a marked improvement of maintaining good electrical conduction for charge transfer by accommodating the S cathode.

**Fig. 5:**
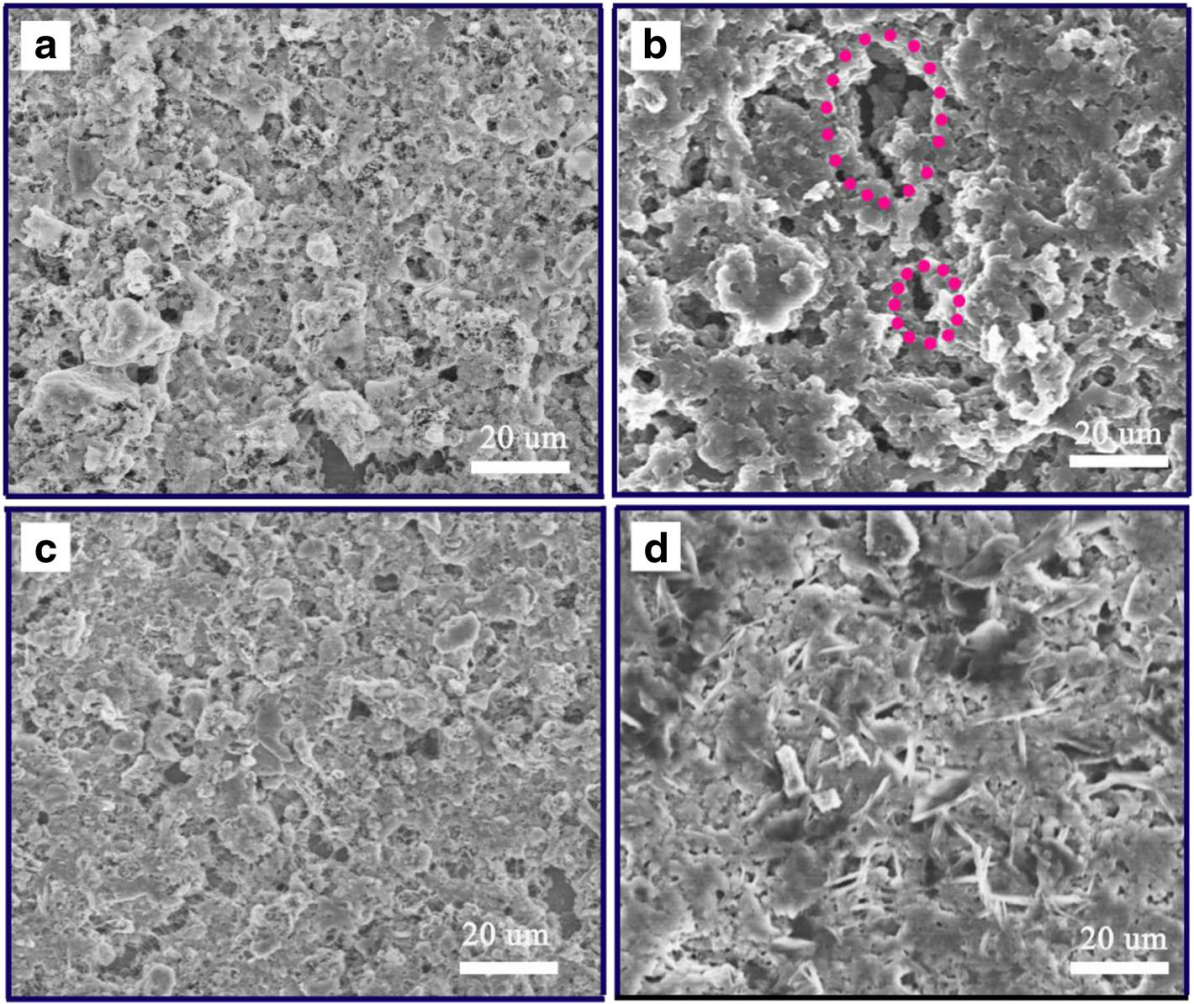
SEM images of fresh electrodes: **a** S@PVDF, **c** S@AHP and of the electrodes after 50 cycles: **b** S@PVDF, **d** S@AHP electrodes

To further investigate the mechanical stability of C/S electrodes, C/S eletrodes were prepared with 15% AHP and PVDF as binder, respectively. The surface morphologies of different electrodes were characterized by SEM and shown in Fig. 5. Before cycling, no significant differences between the sulfur electrodes with different binders were observed, the active sulfur composite and acetylene black can be clearly distinguished in each electrode, indicating the AHP binder can effective bond the active materials. Differently, AHP binder presents an example of a more uniformly coated electrode in Fig. 5c. In particular, binder “bridges” emerge of the AHP binder between the adjacent C/S materials can be observed, indicating that the AHP binder has sufficient capacity to connect the active materials. In addition, the coated film has a strong adhesion to the Al foil. No materials peel off during the subsequent operations in which the electrode is bended and folded repeatedly. After 50 deep galvanostatic discharge at 0.5 C, Both S@AHP and S@PVDF cathodes exhibited uniform morphology distributions (Fig. 5b, d) as well as similar SEI formation filling the void space after the lithiation. The difference between the samples, however, became evident after 50 cycles such that S@AHP still preserved the uniform morphology distribution to a large extent (Fig. 5d), whereas S@PVDF clearly showed micrometer scale cracks (Fig. 5b, red oval) over the entire area of the film, indicating the AHP binder is better at preserving the original film morphology utilizing its superior multidimensional binding capability based on active site of amide groups [39]. These results clearly demonstrate that the polar AHP binder has capacity to maintain the electrical and mechanical integrity of S based cathodes upon deep galvanostatic cycling.

## Conclusions

In summary, we have successfully developed a polar binder with plenty amide groups as multidimensional bonding site for high-performance Li-S cells, making substantial progress in improving electrochemical properties and therefore resolving the chronic insufficient cycle lives of S cathode. We demonstrate the lots of amide functional groups of the AHP binder with high binding strength construct effectual trap the sulfur species and subsequently confine them within the cathode and inhibit the shuttling effect, while the excellent mechanical properties of the S@AHP cathode with suitable flexible to buffer the volume change of sulfur. When AHP was applied to assemble cells with commercial sulfur and acetlene black have been cycled, they could show the stable capacity retention at different rates of cycles. As a result, we believe the synthesis of this polymeric polymer will arouse the battery community’s interest in fabricating long life Li-S cells and provide a novel method for synthesis new materials for Li ion batteries.
